# Role of IL-6 gene polymorphisms in children with autism spectrum disorders

**DOI:** 10.1186/s13052-026-02245-2

**Published:** 2026-04-11

**Authors:** Noha M. Abdullah, Hend M. Moness, Marwa Waly Eldin Ali, Ebtihal Mokhtar Abdelsamei, Ebtesam Esmail Hassan, Dalia Abdelrahman Meshref

**Affiliations:** 1 Clinical and Chemical Pathology, Faculty of Medicine, Mina University Hospital, Minia, Egypt; 2 Faculty of Medicine, Pediatric Department Mina University Hospital, Minia, Egypt; 3 Public Health and Preventive Medicine, Faculty of Medicine, Minia University, Minia, Egypt

**Keywords:** ASD, IL-6 gene polymorphisms, IL-6, GARS-3

## Abstract

**Background:**

Autism spectrum disorder (ASD) is a common neurodevelopmental disorder characterized by impairment in social interaction, and communication skills, along with restricted or repetitive behaviors. The diagnosis of ASD depends on behavioral parameters. Numerous studies have reported immune system abnormalities and proposed a potential role of autoimmunity in the pathogenesis of ASD. This study aims to assess the correlation between specific cytokines, such as interleukin-6 (IL-6), and particular IL-6 polymorphisms, including IL-6-174G/C (rs1800795) gene polymorphism, IL-6–572 C/G (rs1800796) gene polymorphism and IL-6-597G/A (rs1800797) gene polymorphism among children with ASD.

**Methods:**

The current study included 100 children with ASD were recruited during their regular follow up to Pediatric Neuro-Psychiatry Clinic of Minia University Hospital of Children. They diagnosed according to DSM-5 ASD criteria. A another 100 children were recruited as control group by simple randomly selected school (pre & elementary school) in Mina Governate, Upper Egypt, their ages and sex matched with the ASD children, they were apparently neuropsychiatric and developmentally normal and free from any chronic systemic illness. The participants were assessed for serum IL-6 levels and single-nucleotide changes in IL-6-174G/C, IL-6–572 C/G and IL-6-597G/A gene polymorphisms.

**Results:**

The prevalence of the GG genotype and G allele of the IL-6-174G/C gene polymorphism was significantly higher in ASD patients compared to healthy control (P value = 0.0002, 0.03 respectively). In contrast, the GC genotype and C allele of IL-6 -174G/C gene polymorphism were significantly elevated in the control group compared to children with ASD, indicating a protective role against ASD in the Egyptian population. The prevalence of the CG genotype, GG genotype, and G allele of the IL-6–572 C/G gene polymorphism was significantly higher in ASD patients compared to control individuals, indicating an increased risk of ASD within the Egyptian population. The IL-6-597G/A gene polymorphism analysis revealed no significant differences between the two groups in GG and GA genotypes or allelic frequencies. Nevertheless, the AA genotype was only found in the autistic group. Furthermore, ASD patients exhibited significantly higher serum levels of IL-6 than the healthy controls. The GG genotype distribution of IL-6-174G/C gene polymorphism was significantly associated with increased serum levels of IL-6.

**Conclusion:**

The IL-6 (-174G/C) gene polymorphism (GG genotype) was associated with ASD. Moreover, the (CG + GG) genotypes of IL-6–572 C/G gene polymorphism were associated with ASD, and the (AA genotype) of IL-6-597G/A gene polymorphism was detected only in ASD. This indicates their role in the increased incidence of ASD among the Egyptian population. In addition, IL-6 serum level was significantly elevated in ASD.

**Supplementary Information:**

The online version contains supplementary material available at 10.1186/s13052-026-02245-2.

## Background

ASD is a complex neurodevelopmental disorder that manifests in early childhood. ASD is typically associated with lifelong cognitive, communicative, social, and behavioral impairments in individuals [1]. These symptoms result from complex interactions of risk factors during pre- and post-natal brain development [2]. The number of individuals diagnosed with ASD has significantly increased dramatically over the past 40 years. The Centers for Disease Control and Prevention (CDC) reported a significant rise in diagnosed cases from 1 in 150 in 2000 to 1 in 36 in 2020 [3, 4]. The prevalence of ASD in Egypt ranges from 5.4/1000 to 33.6% [5]. Furthermore, research conducted in Egypt was restricted to specific regions, was facility-based, and involved a small sample size [6, 7]. The precise pathogenesis of Autism Spectrum Disorder (ASD) remains undetermined. However, it is recognized as multifactorial. Both maternal and embryonic genetic mutations may interact with external factors, including toxins, infections, nutritional deficiencies, environmental pollutants [8], and internal factors, such as autoimmune diseases [9]. These interactions can activate microglia and trigger the release of various cytokines, particularly interleukin 6 (IL-6), which adversely affect the structure and function of the developing brain [10, 11]. The predominant mechanism linked to ASD was immune dysfunction and inflammation. Stress, autoimmune diseases, infections, and dysbiosis were found to facilitate the release of pathogen-associated molecular patterns and damage-associated molecular patterns, which bind to toll-like receptors on maternal leukocytes and placental cells, leading to maternal immune activation (MIA). This activation results in an elevation of IL-6 concentration, which stimulates T helper 17 lymphocytes that promote the release of proinflammatory cytokines, thereby inducing inflammation. Additionally, IL-6 suppresses the activity of immunosuppressive regulatory T-cells [12].

Interleukin (IL)-6 is a crucial neuroimmune factor implicated in physiological brain development and several neurological disorders. For instance, findings from postmortem and animal studies suggest that brain IL-6 is an important mediator of autism-like behaviors [13]. Moreover, modified T cell and monocyte immune responses have been identified in ASD, potentially linked to behavioral modulation and core features in individuals with ASD [14, 15]. Numerous studies indicated that certain IL-6 gene polymorphisms are associated with increased risks for the development of ASD [16]. The IL-6 gene contains a single nucleotide polymorphism (SNP) located in the chromosomal region 7p21.1–22.3, and prior research indicated that the IL-6–572 C/G gene polymorphism may affect the transcriptional rate of the IL-6 gene and the plasma concentrations of acute inflammatory proteins, including fibrinogen and C-reactive protein. The extent of cytokine production is contingent upon the antigenic challenge and the host’s genetic factors [17]. Therefore, the identification of SNPs of cytokines serves as a promising tool to enhance our understanding of the pathogenesis of ASD, determine probable markers of disease susceptibility and severity, and investigate the association between IL-6 and ASD in conjunction with other inflammatory markers. This study aims to evaluate the relationship between serum interleukin-6 (IL-6), specific IL-6-174G/C, IL-6–572 C/G and IL-6-597G/A gene polymorphisms., and the association of these polymorphisms with ASD severity in children.

## Subjects and methods

### Study design

This study included 100 Children with ASD were recruited during their regular follow-up to the Pediatric Neuro-Psychiatry Clinic of Minia University Hospital of Children in the period from April 2024 to November 2024. The control group was 100 children and they were selected by simple randomly selected school (pre & elementary school) in Mina Governate, Upper Egypt, their ages and sex matched with the ASD children. They are neuropsychiatric and developmentally normal children, without any chronic systemic diseases, or acute inflammatory illness (determined by complete history, general examination and neurological examination), they have no family history of ASD in their extended family. The extended family means a family that includes not only parents and children but also other relatives such as grandparents, aunts, or uncles to exclude any ASD genetic predisposition for the control group [18]. The study protocol was approved by Minia University, Faculty of Medicine, Institutional Review Board (MUFMIRB) (Approval No: 720 = 4=2023). All parents of participants provided informed written consent. The children were diagnosed with ASD by experienced psychologists according to the criteria outlined in the Diagnostic and Statistical Manual of Mental Disorders, Fifth Edition (DSM-V). The severity of symptoms was evaluated using the Gilliam Autism Rating Scale Arabic version (GARS-3), a psychometric tool for diagnosing and establishing a baseline of autistic features for ages 3–22 years. It comprises 56 items, subdivided into subscales, including communication, social interaction, stereotyped behaviors, and developmental scores. A higher score correlates with more severe autistic symptoms [19]. The severity of autism was determined according to (Supplementary Table 1) [20]. ASD Children aged 3–18 years were subjected to a complete medical history (focusing on social isolation and interaction, stereotyping movements, hypo/hyperactivity, motor and sensory symptoms, aggression, self-mutilation and sleep disorders), anthropometric measures, clinical examination and neurological examination. sleep disorders (SD) means any disturbance of normal sleep patterns. The DSM-V divided SD into 10 disorders: insomnia disorders, hypersomnolence disorder, narcolepsy, breathing-related sleep disorders, circadian rhythm sleep–wake disorders, non-rapid eye movement (NREM) sleep arousal disorders, nightmare disorder, rapid eye movement (REM) sleep behavior disorder, restless legs syndrome, and substance/medication-induced sleep disorder. These disorders have disrupted nocturnal sleep or daytime sleepiness as primary sleep complaints and are all associated with impaired daytime functioning) [21]. Children with systemic diseases, acute infection, febrile illness, history of allergy or immunological issues, recent vaccinations with live attenuated or killed vaccines, and neurological or psychiatric disorders other than autism were excluded from the study.

### Sample size

Sample size was calculated using software program G power. Using the results of IL6 genotype polymorphisms [16] at power of 80% and significant level less than 0.05. The sample size will be 95.

### Laboratory investigations

First, 7 ml of venous blood was withdrawn from all subjects. The sample was divided into (1) 1 ml in a sterile vacutainer tube containing EDTA solutions for CBC assay. (2) 2 ml in a sterile vacutainer tube containing EDTA solutions for DNA isolations and assessment of genetic detection. (3)1.6 ml was added to a sterile Trisodium citrate tube containing 0.4 ml of 3.8% TSC solution for ESR determination (4) 2 ml of whole blood was collected in a serum separator gel vacutainer tube for serum separation. The serum was prepared by allowing the whole blood to clot for 30 min at 37 °C, followed by centrifugation at 3500 rpm for 15 min. The serum supernatant was used to determine the serum IL-6 and hs-CRP levels. CBC was performed using the automated cell counter Celltac ES (Nihon Kohden Europe). Subsequently, Hs-CRP was assessed by a specific protein analyzer using the nephelometry method (Shenzhen Genius Electronics, China). Serum IL-6 was assessed via the fluorescence immunoassay method using (BIOT-YG-IFIA immuneanalyzer, China). Finally, ESR was assessed via the Westergren method.

### Determination of IL-6 gene polymorphisms

The detection of IL-6 Single nucleotide polymorphisms (SNPS) was performed by polymerase chain reaction and restriction fragment length polymorphism (PCR-RFLP) analysis. Genomic DNA was extracted from the leukocyte portion of whole blood using (QiaAmp DNA extraction kit, Qiagen, Germany). Nanodrop was then used to assess the quality of the DNA.

DNA was amplified in a 15µL reaction mix made from 0.5 µL extracted DNA, 1 µL of each primer specific for each SNP, 7 µL master mix, and 5.5 µL distilled water. The PCR cycle for the three SNPs was as follows: An initial denaturation step of 95 °C for three minutes, followed by 30 cycles of denaturation at 94 °C for 45 s, an annealing temperature of 30 s at 58 °C for IL-6 − 174G/C gene polymorphism and 20 s at 61 °C for IL-6–572 C/G and IL-6 − 597 G/A gene polymorphism and a final extension step at 72 °C for ten minutes.

Each SNP has a specific RFLP digestion enzyme (restriction enzyme) according to the manufacturer’s instruction kit supplied by (Thermo Fisher Scientific Inc.). BseL-I restriction enzyme was used to digest IL-6 − 174G/C, BsrB-I restriction enzyme was utilized for IL-6–572 C/G lastly Fok-1 restriction enzyme was used for IL-6 − 597 G/A. The fragments’ sizes were obtained using a 2% agarose gel and then analyzed using the Gel-Pro-Analyzer program.

### Statistical analysis

Statistical analysis was conducted using SPSS software version 21.0 graphics (SPSS Inc., Chicago, IL, USA) using Excel. Quantitative variables were reported as mean ± standard deviation (SD), whereas Qualitative data was presented as frequency distribution. An independent sample t-test, one-way ANOVA, and post hoc test were conducted to assess the significant differences in quantitative data. Pearson’s chi-square (χ2) was used to test the significant difference in qualitative data. Regression analysis was done, and odds ratio (OR) ratios and 95% confidence intervals were calculated. P-values < 0.05 were considered statistically significant.

## Results

- **The demographic data and Gilliam autism rating scale (GARS-3) scores of studied autistic children**:

The mean age of autistic children in the present study was 6.50 ± 2.90 years. The majority of the examined autistic children were male (80%), and 80% had a positive familial history of ASD. Their BMI was 18.60 ± 4.50, with 60% exhibiting sensory hyperesthesia and 16% demonstrating sensory hypesthesia. Furthermore, 56% exhibited aggressive behaviors, which may be directed towards caregivers or manifest as self-injurious behaviors. They may exhibit behaviors such as kicking, throwing objects, or self-harm; 56% experienced sleep disturbances characterized by insomnia, difficulties with bedtime settling, sleep anxiety, sleepwalking, poor sleep quality, and challenges in initiating or maintaining sleep. The majority experienced linguistic difficulties. Additionally, 52% exhibited echolalia. The average GARS-3 index score among the studied autistic children was 104.80 ± 19.10, with a range of 70.0–139.0 (indicating mild to above high severity of ASD symptoms). The mean scores for the GARS-3 subscales were 10.20 ± 4.06 for interaction, 11.80 ± 3.30 for communication, and 11.80 ± 3.30 for stereotyping. A majority of the autistic children exhibited average to high severity of ASD symptoms, comprising 40% and 24% of the cohort, respectively (Table 1).

**Table 1:** The demographic data and Gilliam autism rating scale (GARS-3) scores of studied autistic children

Clinical data	Autistic children *N* = 100	Control group *N* = 100	*p*-value
Age (years)	6.50 ± 2.90	5.70 ± 3.70	0.07
Sex			
male	80%	75%	0.30
Female	20%	25%	
Residence			
Urban Rural	44% 56%	50% 50%	0.30
Family history			
Negative% Positive %	20% 80%	100% 0%	0.001*
History of sensory affection			
No sensory affection% Hyper-thesia % Hypo-thesia %	24% 60% 16%	-	-
aggressive behavior			
Negative% Positive%	44% 56%	-	-
Sleep disorder			
Negative% Positive%	44% 56%	-	-
Echolalia			
Negative% Positive%	48% 52%	-	-
BMI kg/m²			
Range Mean ± SD	12.0–30.0 18.60 ± 4.50	17.0–27.0 20.20 ± 3.40	0.008*
GARS-3 scores			
GARS-3 raw total score			
Range Mean ± SD	16.0–48.0 31.70 ± 8.90	-	-
GARS-3 index			
Range Mean ± SD	70.0 -139.0 104.80 ± 19.10	-	-
GARS-3%			
Range Mean ± SD	2.0 -100.0 56.0 ± 33.90	-	-
GARS-3 social interaction			
score Range Mean ± SD	4.0–16.0 10.20 ± 4.06	-	-
communication score			
Range Mean ± SD	6.0–19.0 11.80 ± 3.30	-	-
Stereotyping score			
Range Mean ± SD	6.0–19.0 11.80 ± 3.30	-	-
Autism severity type%			
Low Low average Average Above average High Above high	8% 16% 40% 4% 24% 8%	-	-

b. **Comparison between Autistic children and controls regarding laboratory investigations**:

The complete blood picture revealed a significantly lower hemoglobin concentration in autistic children compared to the control group (P-value = 0.001). Conversely, there were significantly higher levels of total leukocyte count, lymphocyte count, eosinophils, monocytes, monocyte-to-lymphocyte ratio, and platelets (P-values = 0.006, 0.001, 0.02, 0.001, 0.001 and 0.003, respectively) in autistic children than the control group which supports the inflammatory theories of the ASD pathogenesis. The acute phase reactants, specifically serum levels of interleukin 6, hs-CRP, and ESR at both the first and second hours, were significantly elevated in autistic children compared to the control group (p-values = 0.001, 0.001, 0.001, and 0.001, respectively) (Table 2; Fig. 1).

**Table 2:** Comparison between Autistic children and controls regarding laboratory investigations

Data	Autism cases *N* = 100	Controls *N* = 100	*P*-value
Hb (g/dl)			
Range Mean ± SD	10.80–14.10 12.10 ± 0.70	12.0–14.0 12.80 ± 0.60	0.001*
Platelet count (x10^9^/L)			
Range Mean ± SD	228.0–420.0 326.60 ± 60.10	253.0–371.0 301.20 ± 43.20	0.003*
TLC (x10^9^/L)			
Range Mean ± SD	3.30–17.20 7.60 ± 3.04	5.0–7.0 6.30 ± 0.70	0.006*
Lymphocyte (x10^9^/L)			
Range Mean ± SD	15.0–59.0 43.90 ± 10.60	38.0–56.0 49.70 ± 6.90	0.001*
Absolute lymphocyte/L			
Range Mean ± SD	1155.0–8600.0 3346.30 ± 1576.60	2660.0–3696.0 3069.0 ± 356.30	0.80
Neutrophils (x10^9^/L)			
Range Mean ± SD	25.0–78.0 47.20 ± 11.80	41.0–60.0 49.20 ± 11.80	0.10
Eosinophil(x10^9^/L)			
Range Mean ± SD	1.0–10.0 1.88 ± 1.80	1.0–2.0 1.34 ± 0.40	0.02*
Monocyte(x10^9^/L)			
Range Mean ± SD	3.0–10.0 6.40 ± 1.90	1.0–3.0 1.80 ± 0.80	0.001*
Absolute Monocyte /L			
Range Mean ± SD	153.0–1720.0 493.60 ± 294.70	53.0–70.0 62.10 ± 6.03	0.001*
NLR			
Range Mean ± SD	0.43–5.20 1.20 ± 0.90	1.0–2.0 1.10 ± 0.30	0.50
MLR			
Range Mean ± SD	0.10–0.33 0.15 ± 0.06	0.01–0.02 0.01 ± 0.001	0.001*
PLR			
Range Mean ± SD	48.80–322.0 119.30 ± 67.30	92.0–109.0 97.80 ± 5.70	0.20
hs-CRP (mg/L)			
less than 3 mg/L 3–10 mg/L More than 10 mg/L	68.0% 20.0% 12.0%	100(100%) 0 0	0.001*
Interleukin 6 (pg/mL)			
Range Mean ± SD	2.0 -1000.0 56.08 ± 195.03	2.0–7.0 3.80 ± 1.90	0.001*
ESR (mm/hr)			
1st hour mean ± SD 2nd hour mean ± SD	22.30 ± 13.30 38.50 ± 19.80	12.20 ± 1.20 20.10 ± 3.30	0.001* 0.001*

Hb: Hemoglobin, TLC: Total leukocyte count, NLR: Neutrophil Lymphocyte Ratio, MLR: Monocyte lymphocyte Ratio, PLR: Platelet Lymphocyte Ratio, hs-CRP: High Sensitive C- Reactive Protein, ESR: Erythrocyte Sedimentation Rate, * Significant level of p- value is < 0.05

**Fig. 1:**
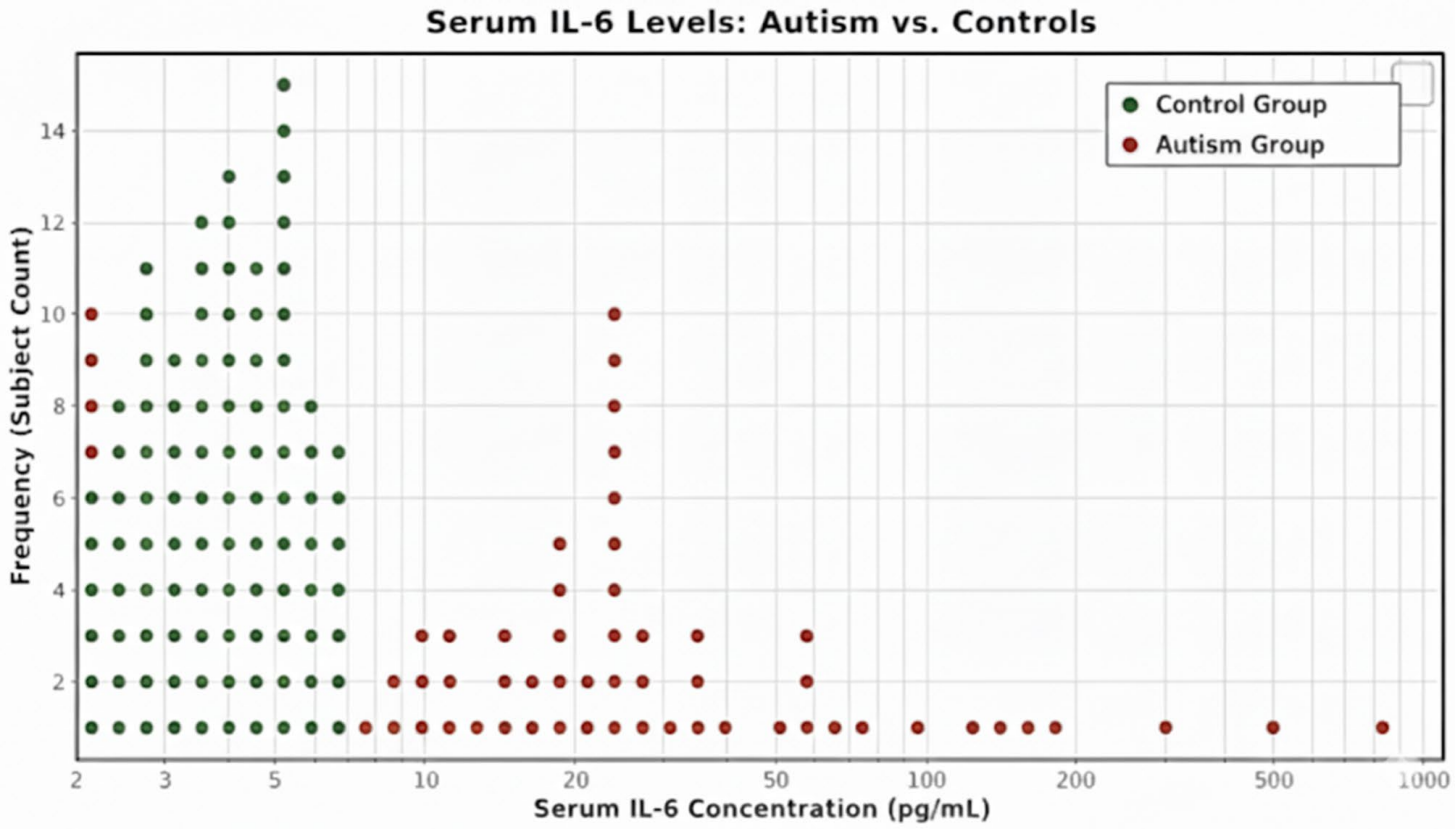
Dot plot distribution of serum Interleukin-6 (IL-6) levels between a control group and Autistic group. The control group represented narrow distribution, between (2.0 and 7.24) pg/mL. While in autistic group shows elevation of IL-6, including extreme outliers reaching 1000 pg/mL. This wide variation among group most likely due to variability in disease severity

c. **Allele frequencies and Genotype distribution of IL-6-174 G/C**,** IL-6–572 C/G and IL-6-597G/A gene polymorphisms among studied children**:

IL-6-174G/C gene polymorphism showed a statistically significant higher frequency of GG genotype in autistic children (60.0%) when compared to the control group (33.0%) (p-value = 0.002). The GC genotype showed a statistically significant increase in frequency in the control group (67.0% (when compared to autistic children (40%) (p-value = 0.002). The CC genotype was not detected in both autistic children and the control group. Interestingly, the C allele showed statistically significantly increased frequency in the control group compared to the autistic children group (33.0% vs. 20.0%) (p value = 0.03). This suggests that the GC genotype and C allele may have a protective role against ASD (OR = 0.39, 95% CI = 0.23–0.65, *p* = 0.001) (OR = 0.34, 95% CI = 0.19–0.61, *p* = 0.001) respectively. In contrast, the GG genotype, the G allele, may be a predisposing factor for ASD within the Egyptian population (OR = 2.90, 95% CI = 1.60–5.10, *p* = 0.001). (OR = 2.03, 95% CI = 1.20–3.20, *p* = 0.001) respectively.

Regarding IL-6–572 C/G gene polymorphism analysis, the CC genotype showed higher statistically significant frequency in the control group (83.0%) when compared to the ASD group (44.0%) (p value = 0.001). While the CG genotype showed highly significant increased frequency in the ASD group (36.0%) when compared to the control group (17.0%) (p-value = 0.002), the GG genotype was detected only in the ASD group (20.0%). The allelic frequency of this polymorphism showed a statistically significant higher frequency of the C allele (91.50%) in the control group than in the autistic group (61.50%) (p-value = 0.001). Conversely, the G allele showed a higher statistically significant frequency in the ASD group (38.50%) than in the control group (8.50%) (p-value = 0.001). Therefore, both CG and GG genotypes were associated with an increased risk of ASD (OR = 2.70, 95% CI = 1.40–5.30, *p* = 0.001) (OR = 2.20, 95% CI = 1.90–2.60, *p* = 0.001) respectively. In addition, the G allele was associated with an increased risk of ASD (OR = 6.70, 95% CI = 3.80–11.90), *p* = 0.001) while CC genotype and C allele may have protective role against ASD (OR = 0.16, 95% CI = 0.08–0.31, *p* = 0.001), (OR = 0.14, 95% CI = 0.08–0.26, *p* = 0.001) respectively.

**Fig. 2:**
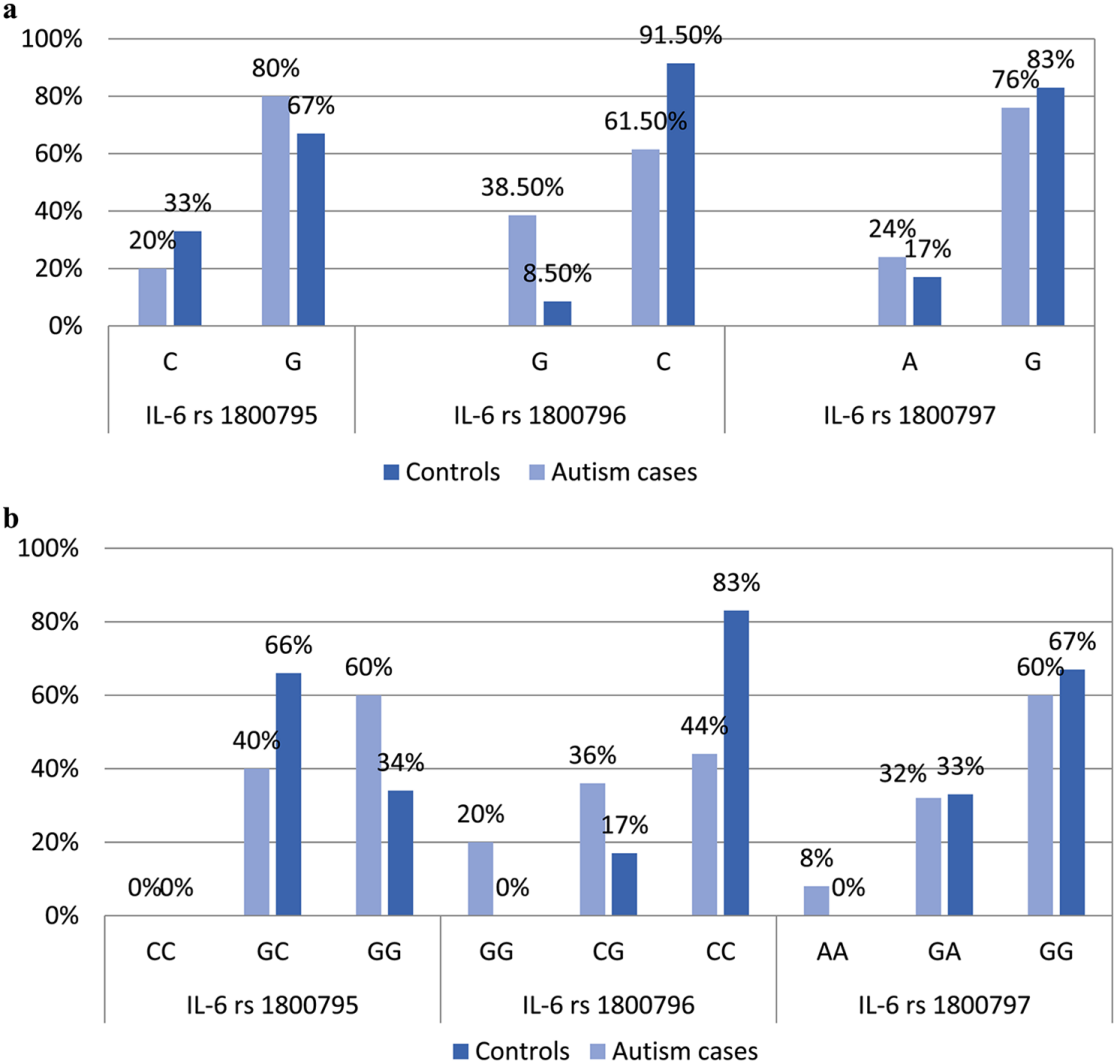
shows Allelic and genotype frequencies of studied IL-6 SNPs among studied groups. **a**: Allelic frequencies of studied IL-6 SNPs among studied groups and **b**: Genotype frequencies of studied IL-6 SNPs among studied groups

In contrast, the analysis of IL-6 − 597G/A gene polymorphism. The GG and GA genotypes exhibited no significant difference between the two groups (p-values = 0.30 and 0.70, respectively). However, the AA genotype was only found in the autistic group (8.0%) with odd ratio (OR = 2.08, 95% CI = 1.80–2.40, *p* = 0.01) so it was associated with increased risk of autism. There was no significant difference in allelic frequencies between both groups (p-value = 0.20) (Table 3; Fig. 2).

**Table 3:** Allele frequencies and Genotype distribution of IL-6-174 G/C, IL-6–572 C/G and IL-6-597G/A gene polymorphisms among studied children

SNP	Alleles/genotypes	Autism cases *N* = 100	Controls *N* = 100	*P*-Value	OR (95% CI)	*P*-value for odds ratio
IL-6-174 G/C	Genotypes					
	GG GC C	60 (60.0%) 40 (40.0%) 0	33 (33.0%) 67 (67.0%) 0	0.002* 0.002*	2.90 (1.60–5.10) 0.34 (0.19–0.61)	0.001*
	Alleles					
	G C	160(80.0%) 40 (20.0%)	134(67.0%) 66 (33.0%)	0.03* 0.03*	2.03 (1.20–3.20) 0.39 (0.23–0.65)	0.001*
IL-6–572 C/G	Genotypes					
	CC CG GG	44 (44.0%) 36 (36.0%) 20 (20.0%)	83 (83.0%) 17 (17.0%) 0	0.001* 0.002* 0.001*	0.16 (0.08–0.31) 2.70 (1.40–5.30) 2.20 (1.90–2.60)	0.001*
	Alleles					
	C G	123(61.50%) 77 (38.50%)	183(91.50%) 17 (8.50%)	0.001* 0.001*	0.14 (0.08–0.26) 6.70 (3.80–11.90)	0.001*
IL-6-597G/A	Genotypes					
	GG GA AA	60 (60.0%) 32 (32.0%) 8 (8.0%)	66 (66.0%) 34 (34.0%) 0	0.30 0.70 0.003*	0.77 (0.43–1.30) 0.91 (0.50–1.60) 2.08 (1.80–2.40)	0.01*
	Alleles					
	G A	152 (76.0%) 48 (24.0%)	166 (83.0%) 34 (17.0%)	0.20 0.20	0.64 (0.39–1.06) 1.5 (0.94–2.50)	0.08

OR: Odds Ratio, * Significant level of p- value is < 0.05

d. **Relations between IL-6-174 G/C)**,** IL-6–572 C/G and IL-6-597G/A gene polymorphisms and Gilliam autism rating scales scores in autistic children**:

Regarding IL-6-174G/C gene polymorphism, no significant associations were observed between this polymorphism and the GARS 3 total score, index, communication, social interaction, or stereotyping score (p-values = 0.90, 0.70, 0.90, 0.30, 0.10 respectively). There were significantly higher GARS3 total score, index and stereotyping score in CG Allele of IL-6–572 C/G polymorphism than other studied alleles (p-values = 0.007, 0.04, 0.001, respectively) while higher communication problems in GG than other alleles (p value = 0.009). Nonetheless, no significant differences were observed among AA, GA, and GG genotypes of IL-6 − 597G/A gene polymorphism in all assessed GARS-3 scores, except for a significantly higher GARS − 3 stereotyping score in the GA allele (p-value = 0.001) (Table 4).

**Table 4:** Relations between IL-6-174 G/C, IL-6–572 C/G and IL-6-597G/A gene polymorphisms and Gilliam autism rating scales scores in autistic children

GARS-3 score Mean ± SD	IL-6-174 G/C gene polymorphism			IL-6-174 G/C gene polymorphism				IL-6-174 G/C gene polymorphism			
	GG (60)	GC (40)	*p*-value	CC (44)	CG (36)	GG (20)	*p*-value	GG (60)	GA (32)	AA (8)	*p*-value
Social Interaction	10.50 ± 4.20	9.70 ± 3.70	0.30	9.90 ± 4.10	9.70 ± 3.60	11.60 ± 4.50	0.20	10.06 ± 3.90	10.20 ± 4.70	10.70 ± 0.40	0.90
Communication	9.70 ± 4.30	9.80 ± 1.80	0.90	8.50 ± 3.70	10.60 ± 3.02	10.80 ± 3.20	0.009*	9.30 ± 3.60	10.80 ± 3.60	8.50 ± 0.50	0.08
Stereotyping	11.40 ± 2.80	12.40 ± 3.80	0.10	10.10 ± 2.20	14.0 ± 3.30	11.40 ± 3.01	0.001*	11.50 ± 2.60	13.20 ± 3.90	8.20 ± 4.70	0.001*
Gilliam Autism Index	105.30 ± 20.60	104.0 ± 16.80	0.70	107.80 ± 17.80	109.50 ± 18.10	99.50 ± 19.60	0.04*	103.80 ± 17.90	109.50 ± 22.20	93.50 ± 2.60	0.08
Gilliam Total Score	31.60 ± 9.60	31.80 ± 7.80	0.90	33.60 ± 8.30	34.40 ± 8.40	28.60 ± 8.70	0.007*	30.90 ± 8.30	34.30 ± 10.30	27.0 ± 1.06	0.06

* Significant level of p- value is < 0.05

e. **Relations between IL-6-174G/C**,** IL-6–572 C/G and IL-6-597G/A gene polymorphisms and laboratory data among autistic children**:

The current study revealed that the GG genotype of **IL-6**-174G/C gene polymorphism is associated with significantly elevated levels of platelet count, TLC, absolute lymphocyte count, eosinophil count, monocytic count, absolute monocytic count, MLR, PLR, IL-6, and high sensitive C- reactive protein (hs-CRP) compared to the CG genotype (P-values = 0.001, 0.01, 0.04, 0.01, 0.01, 0.008, 0.01, 0.05, 0.004, 0.009, respectively) (Table 5).

**Table 5:** Relation between IL-6-174 G/C gene polymorphism and laboratory data in autistic children

Data Mean ± SD	IL-6-174 G/C gene polymorphism				
	GC (40)		GG (60)	*P*-value	
Hb (g/dl)	12.60 ± 0.80		12.20 ± 0.70	0.001*	
Platelet (x10^9^/L)	301.90 ± 56.50		32.50 ± 47.20	0.001*	
TLC (x10^9^/L)	6.61 ± 1.40		7.45 ± 2.90	0.01*	
Lymphocyte (x10^9^/L)	47.30 ± 9.10		46.30 ± 9.70	0.40	
Absolute lymphocyte /L	3056.60 ± 672.80		3378.60 ± 1491.90	0.04*	
Neutrophils (x10^9^/L)	49.50 ± 8.40		47.09 ± 10.80	0.07	
Eosinophil (x10^9^/L)	1.30 ± 0.60		2.20 ± 2.10	0.01*	
Monocyte (x10^9^/L)	3.20 ± 3.05		4.20 ± 2.90	0.01*	
Absolute Monocyte /L	225.20 ± 239.30		337.30 ± 348.10	0.008*	
NLR	1.20 ± 0.40		1.20 ± 0.90	0.70	
MLR	0.07 ± 0.80		103 ± 0.08	0.01*	
PLR	102.26 ± 25.50		115.70 ± 65.003	0.05	
Interleukin 6 (pg/mL)	3.50 ± 2.60		59.80 ± 200.60	0.004*	
ESR (mm/hr)					
1st H 2nd H	22.30 ± 12.70 39.50 ± 19.80	22.30 ± 13.70 37.90 ± 19.90			0.90 0.60
hs-CRP (mg/L)					
less than 3 3–10 More than 10	32(80.0%) 8(20.0%) 0(0%)	36(60.0%) 12(20.0%) 12(20.0%)			0.009*

Hb: Hemoglobin, TLC: Total leukocyte count, NLR: Neutrophil Lymphocyte Ratio, MLR: Monocyte lymphocyte Ratio, PLR: Platelet Lymphocyte Ratio, hs-CRP: High Sensitive C- Reactive Protein, ESR: Erythrocyte Sedimentation Rate, * Significant level of p- value is < 0.05

In the IL-6–572 C/G gene polymorphism gene polymorphism, the CG allele has higher TLC and absolute lymphocytic count than the GG and CC alleles (p values = 0.001, 0.01, respectively). In contrast, the GG allele demonstrates increased monocyte count, absolute monocyte count, and MLR relative to CG and CC alleles (p-valus = 0.001) (Table 6). Finally, the GG allele of IL-6-597G/A gene polymorphism has higher significant values with platelet count, neutrophils, NLR, and PLR (p value = 0.005, 0.04, 0.01, 0.007, respectively). The GA allele exhibits significant increases in absolute lymphocyte and eosinophil counts (p values = 0.02, 0.001), whereas the AA allele demonstrates significantly elevated monocyte and MLR levels (p-values = 0.02, 0.02, respectively) (Table 7).

**Table 6:** relation between IL-6–572 C/G gene polymorphism and laboratory data in autistic children

Data Mean ± SD	IL-6–572 C/G gene polymorphism				Post hoc		
	CC (44)	CG (36)	GG (20)	*P*-value	CC vs. CG	CC vs. GG	CG vs. GG
Hb(g/dl)	12.50 ± 0.70	12.40 ± 0.80	12.40 ± 0.90	0.60	0.30	0.90	0.80
Platelet (x10^9^/L)	312.90 ± 49.20	318.40 ± 58.50	308.40 ± 68.50	0.70	0.50	0.60	0.40
TLC (x10^9^/L)	6.60 ± 1.40	8.02 ± 3.50	6.50 ± 1.90	0.001*	0.001*	0.80	0.01*
Lymphocyte (x10^9^/L)	48.08 ± 9.90	44.50 ± 7.30	45.40 ± 9.90	0.05	0.02*	0.20	0.70
Absolute lymphocyte/L	3115.80 ± 777.80	3564.20 ± 1790.10	2849.2 ± 582.3	0.01*	0.01*	0.30	0.01*
Neutrophils (x10^9^/L)	48.40 ± 10.30	49.0 ± 8.50	46.20 ± 8.90	0.50	0.70	0.30	0.20
Eosinophil (x10^9^/L)	2.20 ± 2.50	1.50 ± 0.60	1.60 ± 0.70	0.10	0.07	0.20	0.80
Monocyte (x10^9^/L)	2.70 ± 2.70	4.70 ± 3.10	7.0 ± 1.70	0.001*	0.001*	0.001*	0.002*
Absolute Monocyte/L	194.50 ± 197.30	402.40 ± 422.40	477.20 ± 253.80	0.001*	0.001*	0.001*	0.30
NLR	1.20 ± 0.80	1.09 ± 0.30	1.10 ± 0.40	0.20	0.09	0.30	0.80
MLR	0.06 ± 0.07	0.10 ± 0.07	0.16 ± 0.07	0.001*	0.001*	0.001*	0.004*
PLR	106.60 ± 38.40	113.30 ± 72.10	112.06 ± 29.70	0.60	0.30	0.60	0.90
Interleukin 6 (pg/mL)	43.20 ± 174.50	6.20 ± 3.40	8.80 ± 7.20	0.20	0.10	0.30	0.90
ESR (mm/hr)							
1st H 2nd H	21.90 ± 13.50 37.30 ± 18.70	22.60 ± 13.20 39.20 ± 20.50	22.60 ± 13.60 40.0 ± 21.30	0.90 0.80	0.80 0.60	0.80 0.60	0.90 0.80
hs-CRP (mg/L)							
less than 3 3–10 More than 10	32(72.70%) 8(18.20%) 4(9.10%)	24(66.70%) 4(11.10%) 8(22.20%)	12(60.0%) 8(40.0%) 0	0.02*	0.20	0.09	0.008*

Hb: Hemoglobin, TLC: Total leukocyte count, NLR: Neutrophil Lymphocyte Ratio, MLR: Monocyte lymphocyte Ratio, PLR: Platelet Lymphocyte Ratio, hs-CRP: High Sensitive C- Reactive Protein, ESR: Erythrocyte Sedimentation Rate, * Significant level of p- value is < 0.05

**Table 7:** Relation between IL-6-597G/A gene polymorphism and laboratory data in autistic children

Data Mean ± SD	IL-6-597G/A gene polymorphism				Post hoc		
	AA (8)	GA (32)	GG(60)	*P*-value	AA vs. GA	AA vs. GG	GA vs. GG
Hb (g/dl)	11.90 ± 0.30	12.50 ± 0.60	12.50 ± 0.80	0.09	0.04*	0.03*	0.80
Platelet (x10^9^/L)	256.50 ± 30.30	311.0 ± 41.10	319.10 ± 58.50	0.005*	0.006*	0.001*	0.30
TLC (x10^9^/L)	6.20 ± 0.50	7.40 ± 2.30	6.80 ± 2.30	0.10	0.10	0.40	0.08
Lymphocyte (x10^9^/L)	44.50 ± 14.40	48.20 ± 6.50	46.30 ± 10.20	0.30	0.20	0.60	0.10
Absolute lymphocyte /L	2691.50 ± 656.90	3501.80 ± 978.60	3086.80 ± 1215.60	0.02*	0.05	0.30	0.01*
Neutrophils (x10^9^/L)	47.0 ± 13.80	46.09 ± 7.80	49.60 ± 10.10	0.04*	0.80	0.40	0.01*
Eosinophil (x10^9^/L)	1.20 ± 0.30	3.0 ± 2.80	1.40 ± 0.40	0.001*	0.008*	0.30	0.001*
Monocyte (x10^9^/L)	6.50 ± 0.50	3.30 ± 2.60	3.70 ± 3.20	0.02*	0.006*	0.01*	0.40
Absolute Monocyte /L	400.0 ± 1.90	256.20 ± 208.90	281.40 ± 345.40	0.40	0.20	0.20	0.50
NLR	1.30 ± 0.60	1.01 ± 0.20	1.30 ± 0.80	0.01*	0.20	0.90	0.003*
MLR	0.15 ± 0.03	0.07 ± 0.06	0.09 ± 0.09	0.02*	0.008*	0.03*	0.10
PLR	103.06 ± 36.50	93.60 ± 22.40	116.70 ± 57.30	0.007*	0.50	0.40	0.004*
Interleukin 6 (pg/mL)	2.50 ± 0.50	14.90 ± 20.70	39.60 ± 175.30	0.40	0.80	0.40	0.20
ESR (mm/hr)							
1st H 2nd H	22.20 ± 13.90 38.10 ± 20.50	22.50 ± 12.40 38.90 ± 18.40	21.70 ± 13.40 40.0 ± 22.05	0.90 0.90	0.90 0.80	0.80 0.80	0.90 0.80
hs-CRP (mg/L)							
less than 3 3–10 More than 10	8(100%) 0 0	20(62.50%) 8(25.0%) 4(12.50%)	40(66.70%) 12(20.0%) 8(13.30%)	0.30	0.10	0.10	0.80

Hb: Hemoglobin, TLC: Total leukocyte count, NLR: Neutrophil Lymphocyte Ratio, MLR: Monocyte lymphocyte Ratio, PLR: Platelet Lymphocyte Ratio, hs-CRP: High Sensitive C- Reactive Protein, ESR: Erythrocyte Sedimentation Rate, * Significant level of p- value is < 0.05

## Discussion

Autoimmune activities accompanied by chronic neuroinflammation have been identified as contributing factors to the etiology of some ASD cases [22]. Many studies demonstrated a significant association between ASD and a family history of autoimmune disease [23]. Moreover, it has been found that increased C-reactive protein during pregnancy in response to IL-6 and other cytokines, such as interleukin-1β and TNF-α, was linked to a high risk of ASD [24, 25]. In our study, we evaluate the relationship between serum IL-6 and specific IL-6 polymorphisms (-174G/C), (-572 C/G) and (-597G/A) and the relation of these polymorphisms with Gilliam autism rating scale that assesses the severity of autism. We found that serum IL-6 was significantly higher in autistic children than in control (p-value = 0.0001). Similarly, a study found that significantly higher levels of serum IL-6 characterized ASD males than healthy controls [26, 27]. Moreover, Systemic maternal inflammation promotes ASD via IL-6 and IFN-γ [28]. Tsilioni et al. observed a significant reduction in serum IL-6 and TNF levels (P-values = 0.036 and 0.015, respectively) at the conclusion of the luteolin formulation treatment compared to baseline levels. The reductions were significantly correlated with the enhancement of children’s behavior post-treatment [29]. Another study recorded that activation of TLR4, CD14 + monocytes from autistic children led to increased production of IL-6 compared to monocytes from children with typical development. IL-6 was also associated with the exacerbation of restrictive and repetitive behaviors [30]. Regarding tryptophan metabolism, IL-6 induces indolamine 2–3 dioxygenase (IDO) enzyme, which plays a key role in the kynurenine pathway (KP) [31]. Children with ASD exhibited elevated serum levels of kynurenic acid, kynurenine, and interleukin-6. These biomarkers are recommended to be assessed in ASD cases as they may be important for the diagnosis of ASD [32]. Another study that was conducted among Korean children contradicts our findings. This outcome demonstrates a distinctive cytokine expression profile in Korean children, characterized by a diminished level of IL-6, attributed to the attenuated protective effects of IL-6 in children with ASD [33].

Our study indicated that the GG genotype of IL-6-174G/C gene polymorphism, the CG and GG genotypes of IL-6–572 C/G gene polymorphism, and the AA genotype of IL-6-597G/A gene polymorphism are correlated with an increased risk of ASD. Limited research has identified a correlation between particular IL-6 polymorphisms and the risk of ASD. Pekkoc Uyanik et al. demonstrated that IL-6 rs1800796 polymorphism presented an elevated risk for the development of ASD among Turkish children with CG genotype and dominant model (CG + GG vs. CC), CG + GG carriers (OR = 1.867, *p* = 0.057; OR = 1.847, *p* = 0.055, respectively) [16]. Han Y et al. found that The IL-6–572 C/G gene polymorphism genotypes may be associated with increased ASD and myelin damage in autistic Chinese children [34]. These studies were in line with our findings. However, there were no studies for detecting IL-6-174G/C and IL-6-597G/A gene polymorphisms. For IL-6-174G/C gene polymorphism, we are the first study that revealed an increase in the frequency of GG genotype in autistic children (60%) (p-value = 0.002). The GC genotype showed a statistically significant increase in frequency in the control group (67.0% (when compared to autistic children (40.0%) (p-value = 0.002). Additionally, the C allele showed a statistically significant increase in the control group compared to the autistic children group (33.0% vs. 20.0%) (p-value = 0.03). Suggesting that the C allele may have a protective role against ASD (OR = 0.39, 95% CI = 0.23–0.65, *p* = 0.001). In contrast, the GG genotype, specifically the G allele, may be a predisposing factor for ASD among the Egyptian population. The GG genotype and G alleles of the IL-6-174G/C polymorphism have been linked to improved transcription of interleukin 6 in several studies [35], which elucidates our findings. The current study is distinctive in demonstrating the AA genotype for IL-6-597G/A gene polymorphism in autistic children, occurring in 8% of cases. Consequently, research involving larger cohorts should be conducted to examine a more substantial correlation between IL6 gene variations and the development of ASD.

Concerning the GARS-3 total score, index, communication, social interaction, and stereotyping score, the CG allele of IL-6–572 C/G gene polymorphism exhibited significantly higher total scores, index, and stereotyping scores than other alleles. Conversely, the GG allele demonstrated elevated social interaction scores and communication problems relative to other alleles. Additionally, no significant differences were observed among the AA, GA, and GG alleles of IL-6-597G/A gene polymorphism across all GARS-3 scores, except for a significantly higher stereotyping score in the GA allele. Contrary to these findings, Han Y et al. discovered that serum levels of IL-6 exhibited positive correlations with the severity of ASD symptoms and the total CARS score. The IL-6–572 C/G gene polymorphism genotype was linked to markedly elevated serum levels of IL-6, yet it did not affect the risk or symptom severity of ASD [34].

According to laboratory investigations, there were significantly lower hemoglobin concentrations in autistic children than in the control group (P-value = 0.001). In contrast, there were significantly higher total leucocytic count, platelet count, lymphocytic count, Eosinophil, Monocyte, and Monocytic lymphocytic ratio (MLR) in autistic children than in the control group, which supports the inflammatory theories of the ASD pathogenesis. Topal Z. et al. also support the involvement of inflammation in the underlying pathophysiology of ASD and other neurodevelopmental disorders, but they found that the neutrophil levels and NLR were higher in the ASD groups and significantly correlated with social interaction problems in ASD [36]. Ferencova N et al. also showed that ASD has higher levels of TLC than overall immune system cells, which are predominantly associated with acute inflammation [26]. Tural Hesapçıog˘lu et al. concluded that increased monocytes, RDW, and decreased lymphocyte-to-monocyte ratio (LMR) are the most obvious findings in ASD. The severity of the disease is associated with decreases in the lymphocyte count and LMR [37]. Moreover, increased monocytes may be due to chronic activation of microglia and monocytes which affect the brain development and function. ASD was showing increased levels of pro-inflammatory cytokines as IL-1B, IL-6 and interferon gamma (IFN- ɣ) resulting from activation of these cells. Additionally, the initial characteristic of acute inflammation is an increase in the levels of neutrophil, chronic inflammation is usually associated with an increase in the levels of mononuclear cells, including monocytes [38–40]. Regarding to eosinophil, previous studies of pediatric neurodevelopmental disabilities (NDDs) revealed mutations on the gene that producing adenosine deaminase enzyme (ADA gene) which responsible for activation of immune system. This mutation associated with both ASD and eosinophilia. Another study recorded association between non-allergic eosinophil activation and NDDs; this is due to L-type amino acid transporter LAT1 that essential for activating T helper 2 cells and causing allergic eosinophilic inflammation. So, all these finding, explained differences counts of monocyte, lymphocyte, neutrophil and eosinophil in present study among autistic children than controls [41]. Finally, our study found that MLR, hs-CRP, ESR 1st and 2nd hour were significantly higher in autistic children than control. Khakzad M. et al. found that hs-CRP in children with autism was significantly higher and correlated with autism severity. These findings confirm the role of inflammation in autism [42]. Moreover, Factor R. et al. stated that ESR observations can yield essential information for assessing ASD status in presymptomatic infants and toddlers aged 13 to 24 months [43].

The limitations of our study were the small sample size and confined to an Egyptian population, which limits its applicability to broader and more genetically diverse populations. Future studies should aim to include participants from multiple regions to validate the findings across different ethnic and genetic backgrounds. The measurement of other proinflammatory cytokines and their associations with IL-6, and the genotyping of IL-6 polymorphisms. In addition, the findings detected other genetic variations and epigenetic modifications, as well as their role in the expression of the IL-6 gene.

Lastly, while the study links IL-6 levels and polymorphisms to ASD severity, it does not provide longitudinal data or insights into how these factors might influence disease progression over time. A longitudinal approach could shed light on the dynamic relationship between inflammation and ASD symptoms.

## Conclusion

This study demonstrates the role of IL-6 heterogeneity in the susceptibility and progression of ASD. The IL-6-174G/C) gene polymorphism, GG genotype was correlated with ASD. Additionally, the (CG + GG) genotypes of the IL-6–572 C/G gene polymorphism were associated with ASD.

The AA genotype of the IL-6-597G/A gene polymorphism was detected only in ASD, suggesting its role in the increased incidence of ASD among the Egyptian population. In addition, the IL-6 serum level was significantly elevated in ASD, suggesting a role of immunity in the pathogenesis of the disease.

## Supplementary Information

Below is the link to the electronic supplementary material.

Supplementary Material 1

Supplementary Material 2

## Data Availability

All data generated or analysed during the current study are available from the corresponding author on reasonable request.
