# Supplementary material for: Role of IL-6 gene polymorphisms in children with autism spectrum disorders
Source: Ital J Pediatr. 2026 Apr 11;52:58. doi: 10.1186/s13052-026-02245-2 (PMC13077965; doi:10.1186/s13052-026-02245-2)
Supplement: Supplementary file 2 — Supplementary Material 2 [file 13052_2026_2245_MOESM2_ESM.docx]

**Supplementary Table**

**Table 1: Classification of autism severity**

| **Autism Severty** | **Autism Index** | **Subscale Scores** |
| --- | --- | --- |
| Extreme high | +131 | 19-17 |
| High | 130-121 | 16-15 |
| Above average | 120-111 | 14-13 |
| Average | 110-90 | 12-8 |
| low average | 89-80 | 7-6 |
| Low | 79-70 | 5-4 |
| Extreme low | 69≥ | 3-1 |
