# Supplementary material for: Role of IL-6 gene polymorphisms in children with autism spectrum disorders
Source: Ital J Pediatr. 2026 Apr 11;52:58. doi: 10.1186/s13052-026-02245-2 (PMC13077965; doi:10.1186/s13052-026-02245-2)
Supplement: Supplementary file 1 — Supplementary Material 1 [file 13052_2026_2245_MOESM1_ESM.pdf]

Dear Professor,

We would like to thank you, the editorial board and the reviewers for their valuable comments that greatly improved the overall quality of our manuscript entitled "**Role of IL-6 gene polymorphisms in children with autism spectrum disorders**". We are enclosing the revised manuscript with the changes included in the text. Also, comments of reviewers (in red italics) were outlined below followed by our detailed responses (in regular black font). We hope that you find our responses satisfactory and that the revised manuscript is now suitable for publication in Italian Journal of Pediatrics.

Reviewer 1 comments:

1- The text in Figure 1 is difficult to read, and part of Figure 2(b) is partially obscured, lacking an explanation of the X-axis.

A more direct comparison of figures 1 (a) and (b) would be better. The current figure makes it difficult to see the differences between the two groups. The X-axis, in particular, needs further explanation.

Author response:

Thank you for your comment, we merge two figures (a) and (b) in one figure and improve the chart to understand the concentration levels of IL-6 among studied groups. Also, we wrote more explanation for chart under the figure as following:

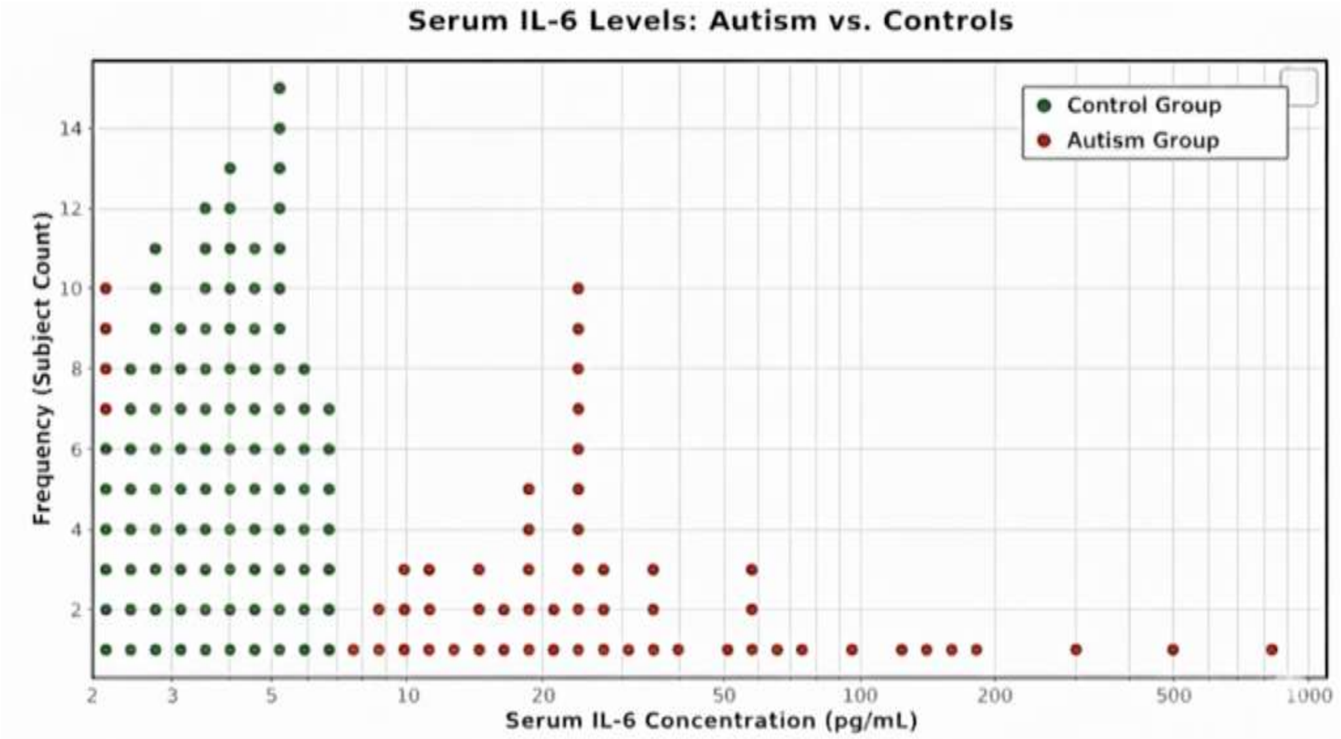

Figure 1 Dot plot distribution of serum Interleukin-6 (IL-6) levels between a control group and Autistic group. The control group represented narrow distribution, between (2.0 and 7.24)pg/mL. While in autistic group shows elevation of IL-6, including extreme outliers reaching 1000 pg/mL. This wide variation among group most likely due to variability in disease severity.

2- Table 1 should include a footnote or other description of the criteria and classification. Furthermore, only "HIGH" is capitalized. The N-number of patients surveyed is required. If the information is simply about classification, it

is irrelevant to the results and should be moved to a supplement or similar document.

Author response:

Thank you for your comment and we moved Table 1 to supplementary file.

3- Please unify the display of total counts and units in Tables 2 through 8.

Author response:

Thank you for your comment and we correct and highlighted them in all tables in the manuscript. Also, as a result of removing table 1 from the previous manuscript, the numbering of tables were changed and become from table 1 to table 7.

4- The fact that the control group had 0% sleep problems (including breathing-related sleep disorders, circadian sleep-wake disorders, and NREM sleep disorders, as described in the Methods section) in Table 2 suggests that the control group differs from our known control group. (In many studies, SRBD occurs in 5-20% of the general population.)

Author response:

Thank you for your comment and let us clarify it. This may be explained by the small sample size, previous study showed significantly higher prevalence of disorders of initiating and maintaining sleep in adolescent girls, ages 11 – 14 years old and snoring in young boys, ages 3 – 6 years old, while the control group in the

current study showed male predominance with mean age 5.70 ± 3.70. Moreover, most of the children with SDB in previous studies have allergic conditions as allergic rhinitis, results in mouth breathing, which in turn leads to impaired maxillofacial development, malocclusion, relative tonsillar enlargement, dysfunction of the genioglossus and geniohyoid muscles, and impaired development of the facial skeleton (typical long face), all predisposing conditions for SDB in the current study children with acute infection, febrile illness, history of allergy or immunological issues were excluded from this study (1-2)

1- Camchildren with hi, S. L., Morgan, W. J., Pernisco, N., & Quan, S. F. (2000). Factors affecting sleep disturbances in children and adolescents. Sleep medicine, 1(2), 117-123.

2- Ng, D.K.; Chan, C.H.; Hwang, G.Y.; Chow, P.Y.; Kwok, K.L. A review of the roles of allergic rhinitis in childhood obstructive sleep apnea syndrome. Allergy Asthma Proc. 2006, 27, 240–242.

5- p.8 and Table 3, Differences in monocyte, lymphocyte, and eosinophil counts are observed, but the explanations for these differences are insufficient.

Author response:

Thank you for your comment, we explained them as following: Moreover, increased monocytes may be due to chronic activation of microglia and monocytes

which affect the brain development and function. ASD was showing increased levels of pro-inflammatory cytokines as IL-1B, IL-6 and interferon gamma (IFN-ɣ) resulting from activation of these cells. Additionally, the initial characteristic of acute inflammation is an increase in the levels of neutrophil, chronic inflammation is usually associated with an increase in the levels of mononuclear cells, including monocytes [38-40]. Regarding to eosinophil, previous studies of pediatric neurodevelopmental disabilities (NDDs) revealed mutations on the gene that producing adenosine deaminase enzyme (ADA gene) which responsible for activation of immune system. This mutation associated with both ASD and eosinophilia. Another study recorded association between non-allergic eosinophil activation and NDDs; this is due to L-type amino acid transporter LAT1 that essential for activating T helper 2 cells and causing allergic eosinophilic inflammation. So, all these finding, explained differences counts of monocyte, lymphocyte, neutrophil and eosinophil in present study among autistic children than controls [41]

6- The units of significant figures within each table should be consistent.

Author response:

Thank you for your comment and we correct them in all tables.

7- In particular, in Tables 6-8, the decimal places of the mean and SD are displayed differently.

Author response:

We apologize and correct them in all tables 5-7.

8- Please also clarify the meaning of PL 0.65.003 and IL6 0.2.6 in Table 6.

Author response:

We apologize for this mistake and we correct them in table 5 as following:

PL 65.003 and IL6 2.60 .
